# Liver X Receptor α (LXRα) Regulates 5β‐Reductase (AKR1D1) Expression in Avian Embryos: Implications for Yolk Steroid Metabolism

**DOI:** 10.1002/jez.70078

**Published:** 2026-02-22

**Authors:** Ryan T. Paitz, Sara E. Waters, Delaney K. Reynolds, Emily M. Drew, Emily P. Harders

**Affiliations:** ^1^ School of Biological Sciences Illinois State University Normal Illinois USA

## Abstract

Steroid‐mediated maternal effects are well‐studied as a source of phenotypic variation. In bird eggs, the yolk contains various steroids that can influence embryonic development. However, one complicating factor in understanding how yolk steroids affect development is that the embryo metabolizes yolk steroids to regulate exposure. The 5β‐reduction of steroids by the enzyme 5β‐reductase (*AKR1D1*) has been identified as a pathway through which yolk progesterone, testosterone, and corticosterone are all metabolized early in avian development. We set out to characterize the mechanism through which *AKR1D1* expression is regulated in chickens (*Gallus gallus*) during embryonic development. We found a synthetic and endogenous ligand (22R‐hydroxycholesterol) for Liver X Receptor α (LXRα) induced *AKR1D1* expression in the embryo and extraembryonic membranes on Day 2 of development. These results suggest that endogenous ligands of LXRα induce *AKR1D1* expression and regulate the metabolism of yolk steroids during development.

## Summary

- During embryonic development, numerous maternally derived steroids in bird eggs are metabolized by the enzyme 5β‐reductase (*AKR1D1*).
- The developing extraembryonic membranes express *AKR1D1* early in development.
- *AKR1D1* expression is induced upon activation of the Liver X Receptor with both synthetic ligands (T0901317) and natural ligands (22R‐hydroxycholesterol).

## 1 Introduction

Vertebrate embryos are sensitive to steroid signals during development, and the effects induced by exposure are often irreversible (Phoenix et al. 1959). Maternally derived steroids have been studied for their potential to program development and influence offspring traits. This “developmental programming” has been implicated in the developmental origins of health and disease in humans (Herbst et al. 1971; Barker 1995), but in wildlife, maternal steroid effects are thought to be beneficial in some contexts (Sheriff et al. 2017; Groothuis et al. 2019). Birds have been well studied when it comes to steroid‐mediated maternal effects, and traits such as growth, immune function, and behavior are influenced by early steroid exposure (reviewed in Groothuis et al. 2005; Henriksen et al. 2011). Decades of work have provided a wealth of examples demonstrating early steroid exposure can affect offspring phenotype in a manner that is potentially adaptive, but the results from these studies have yielded a variety of discrepancies (reviewed in Groothuis et al. 2005; Henriksen et al. 2011. Groothuis et al. 2019; Mentesana et al. 2025). For instance, yolk testosterone has been shown to stimulate embryonic and nestling growth in some bird species (Navara et al. 2005; Muriel et al. 2015), but not in others (Clairardin et al. 2011; Barnett et al. 2011). The possible reasons for these discrepancies have been extensively reviewed and include, but are not limited to, interactive effects between different steroids, embryonic hormone metabolism, and variation in experimental techniques (Podmokła et al. 2018; Groothuis et al. 2019).

To understand how maternal steroids affect development, it is important to know what steroids are present in bird eggs at the time they are laid. While most initial studies on yolk steroids in birds tended to quantify small numbers of steroids (i.e., 1−3 steroids per study), several studies have used liquid chromatography‐tandem mass spectrometry to quantify a larger number of steroids (i.e., 30 steroids) (Merrill et al. 2017; Merrill et al. 2019; Kumar, van Dam, et al. 2019; Hauber et al. 2020; Wang et al. 2023; Enos et al. 2023). From these studies, it is clear that bird eggs contain multiple progestogens, androgens, estrogens, and glucocorticoids. Studies that manipulate steroids at the time the egg is laid to mimic variation in maternal steroid levels demonstrate that avian embryos are capable of responding to many, if not most steroids. For example, increased testosterone boosts offspring growth in numerous species (Groothuis et al. 2005, 2019. However, increased corticosterone inhibits offspring growth in multiple species (Henriksen et al. 2011).

For these responses to occur, it is assumed that steroids travel from the yolk and reach steroid receptors within embryonic tissues to produce the observed effects. Research on steroid receptor expression in avian embryos supports the idea that steroid receptors are present early enough in development to potentially respond to yolk steroids (Kumar, Lohrentz, et al. 2019). Even though maternal steroids are present in the yolk and steroid receptors are present in the embryo, steroid metabolism in the extraembryonic membranes can prevent steroids from activating receptors and eliciting effects (Harders, Charboneau, et al. 2024). Studies on yolk steroid metabolism are thus critical to disentangling when and how maternal steroid effects occur. Some of the earliest work on yolk steroids demonstrated that concentrations of steroids in the yolk decline relatively early in development (Elf and Fivizzani 2002; Eising et al. 2003). It is now known that this decline is the result of steroid metabolism as yolk testosterone (von Engelhardt et al. 2009; Paitz et al. 2011; Kumar et al. 2018; Kumar, van Dam, et al. 2019; Campbell et al. 2020; Wang et al. 2023), progesterone (Paitz and Casto 2012; Paitz and Cagney 2019), estradiol (Paitz et al. 2020), and corticosterone (Vassallo et al. 2014; Vassallo et al. 2019; Harders, Agustin, et al. 2024) are all metabolized in ovo early in embryonic development. From studies identifying specific metabolites during development, it is clear that numerous enzymes are involved in yolk steroid metabolism. In general, yolk steroids are subject to Phase I metabolism (e.g., reduction, hydroxylation) (Paitz et al. 2011; Kumar, van Dam, et al. 2019; Harders, Agustin, et al. 2024) and Phase II metabolism (e.g., sulfonation, glucuronidation) (Paitz et al. 2020; Campbell et al. 2020), and this metabolism results in yolk steroid concentrations rapidly dropping during the early stages of development. In the case of testosterone, etiocholanolone has been identified as a primary metabolite in several bird species (Paitz et al. 2011; Kumar, van Dam, et al. 2019) and the effects of etiocholanolone exposure differ from that to testosterone (Campbell et al. 2020; Wang et al. 2024). Thus, yolk steroid metabolism can alter how maternal steroids influence development.

One enzyme that appears critical to the metabolism of yolk steroids is 5β‐reductase (*AKR1D1*). This enzyme has been studied in bird brains for its ability to metabolize testosterone (Massa et al. 1977; Balthazart and Hirschberg 1979; Soma et al. 2003) and progesterone (Sharp and Massa 1980). It is also involved in the metabolism of yolk testosterone (Paitz et al. 2011; Kumar, van Dam, et al. 2019; Campbell et al. 2020), progesterone (Paitz and Cagney 2019), and corticosterone (Harders, Agustin, et al. 2024). Recent work in chickens has shown that the developing extraembryonic membranes express *AKR1D1*, with levels peaking after just 2 days of incubation (Harders, Agustin, et al. 2024). The primary role of 5β‐reductase is thought to be bile acid production since it is required for conversion of cholesterol into active bile acids (Russell 2003; Chen and Penning 2014). It is likely that bile acids facilitate embryonic absorption of yolk due to its high lipid content. Given the well‐characterized role of 5β‐reductase in bile acid synthesis in the liver, we set out to examine the role of Liver X Receptor α (LXRα or NR1H3) in controlling *AKR1D1* expression (Janowski et al. 1999; Russell 2003). Ligands for LXRα include various endogenous oxysterols (e.g., 22R‐hydroxycholesterol and 24S‐hydroxycholesterol) (Janowski et al. 1999), as well as synthetic ligands like T0901317 (Schultz et al. 2000). Together, these ligands provide a mechanism through which increased levels of cholesterol metabolites activate LXRα to induce the liver enzymes necessary to produce bile acids from cholesterol (which includes 5β‐reductase). Here is we ask the question of whether *AKR1D1* is regulated by *LXRα*? Since *LXRα* regulates bile acid synthesis and *AKR1D1* is required for bile acid synthesis, we hypothesized that *LXRα* regulates *AKR1D1* expression. We predict that activation of *LXRα* with exogenous ligands will increase *AKR1D1* expression.

## 2 Methods

Three studies were conducted to test our hypothesis. Our first study to test this hypothesis used a synthetic ligand for *LXRα* because endogenous ligands for *LXRα* are not well characterized in chickens (*Gallus gallus*). Upon finding the synthetic ligand for *LXRα* was capable of inducing *AKR1D1* expression, the second study tested a common endogenous ligand for *LXRα* (22R‐hydroxycholesterol). The final study tested a range of 22R‐hydroxycholesterol concentrations to identify the minimal dose capable of induction and the maximum levels of induction. All studies used chicken (*G. gallus*) eggs purchased from the University of Illinois Poultry Farm, so the identity of hens was not known. Eggs were collected on a single day for each study, so it can be assumed hens were not contributing more than one egg to any single study. The synthetic LXRα ligand (T0901317) and 22R‐hydroxycholesterol were purchased from Cayman Chemical (Ann Arbor, MI, USA). All manipulations take place prior to the initiation of incubation. After 2 days of development, we collected the central area pellucida and the outer area opaca (Hamburger and Hamilton 1951; Lee et al. 2025). These areas are comprised of the outer ectoderm layer adjacent to the vitelline membrane, the inner endoderm layer adjacent to the yolk, and the middle mesoderm layer that is split in two by the extraembryonic celom (Baggott 2001; Lee et al. 2025). These layers develop into the embryo proper, as well as the amnion, chorion, allantois, and yolk sac (collectively referred to as the “extraembryonic membranes”) (Baggott 2001). Day 2 of development was chosen because *AKR1D1* levels peak at this stage (Harders, Agustin, et al. 2024). In the first study, we compared *AKR1D1* expression in control eggs (vehicle only) to those injected with T0901317. Control eggs (*n* = 13) were injected with 10 µL of vegetable oil, while treated eggs (*n* = 9) were injected with 50 µg of T0901317 dissolved in 10 µL of oil. All injections were done by poking a small hole in the narrow end of the egg with a 16‐gauge needle, injecting vehicle into the albumen, and then sealing the hole with super glue (Henkel Corporation, Rocky Hill, CT, USA) (Campbell et al. 2020; Paitz and Cagney 2019; Paitz et al. 2020; Harders, Charboneau, et al. 2024; Harders, Agustin, et al. 2024). After 2 days of development, samples of the embryo with the extraembryonic membranes were collected and frozen in Trizol (Invitrogen, Carlsbad, CA, USA) until RNA could be extracted for the purpose of qPCR. In the second study, we replicated the first study but added a treatment group where 10 eggs were injected with 40 µg of 22R‐hydroxycholesterol dissolved in 10 µL of vegetable oil. Finally, for the third study, we did a dose curve with 22R‐hydroxycholesterol where eggs (*n* = 8−11 per group) were injected with 0, 10, 50, or 100 ug of 22R‐hydroxycholesterol dissolved in 10 µL of vegetable oil and samples were collected after 2 days of development.

*AKR1D1* expression was quantified using qPCR using the same technique as Harders, Agustin, et al. 2024. Briefly, RNA was extracted from the Trizol reagent according to the manufacturer's instructions and converted to cDNA using a Thermo Scientific Maxima First Strand cDNA Synthesis Kit with Dnase (#K1671). qPCR was performed in triplicate with PowerUp SYBR Green master mix (Applied Biosystems, Waltham, MA, USA) as the indicator and relative expression to the reference gene, *GAPDH*. Efficiencies for *AKR1D1* and *GAPDH* primers are 96% and 98% respectively, so normalized gene expression was calculated as 2^−ΔCT^ (Livak and Schmittgen 2001). We tested for treatment effects in each study using ANOVA with the respective treatments included as fixed effects. One‐way ANOVAs were run using proc GLM in SAS (SAS Institute Inc., Cary, NC, USA). All analyses were run on log‐transformed data to meet the assumptions of ANOVA. For studies 1 and 2, treatment was the sole fixed factor in the model. For study 3, dose was the sole fixed factor in the model.

## 3 Results

**Figure 1:**
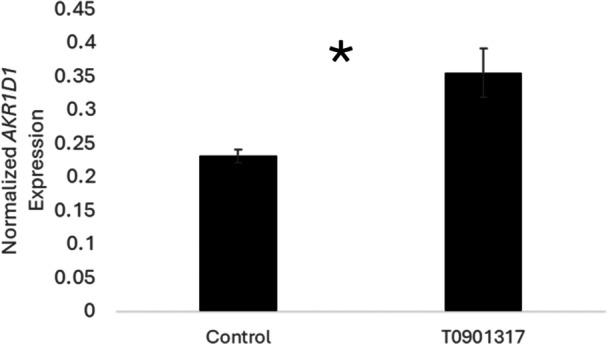
Synthetic LXRα ligand induces AKR1D1 expression. Normalized expression of AKR1D1 in the embryo plus extraembryonic membranes on Day 2 of development is significantly elevated in eggs treated with T0901317 (*p *= 0.006).

**Figure 2:**
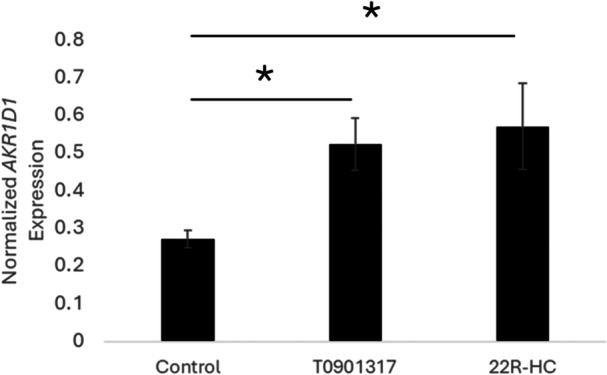
Endogenous LXRα ligand also induces AKR1D1 expression. Normalized expression of *AKR1D1* in the embryo plus extraembryonic membranes on Day 2 of development is significantly elevated in eggs treated with T0901317 (*p* = 0.0009) and 22R‐hydroxycholesterol (22R‐HC) (*p*= 0.002).

**Figure 3:**
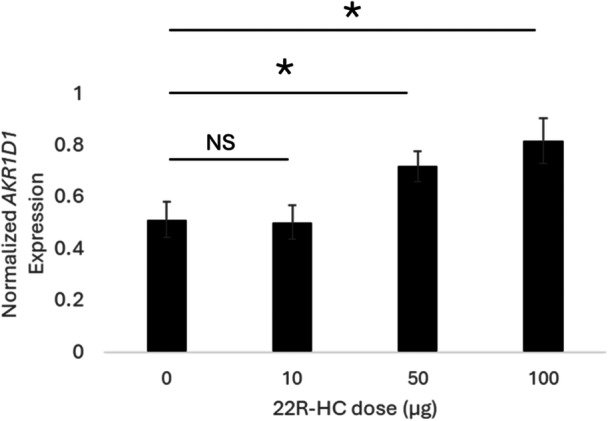
Range of 22R‐HC doses that induce AKR1D1 expression. Normalized expression of AKR1D1 in the embryo plus extraembryonic membranes on Day 2 of development is significantly elevated in eggs treated with 50 and 100 μg of 22R‐HC.

In the first study, *AKR1D1* expression was significantly higher in embryo/extraembryonic membranes from T0901317‐treated eggs compared to control eggs (*F*_1, 20_ = 9.56, *p* = 0.006) (Figure 1). In the second study, we replicated this effect of T0901317 on *AKR1D1* expression and also found that 22R‐hydroxycholesterol also induced *AKR1D1* expression on Day 2 of development (*F*_2, 36_ = 9.56, *p* = 0.001) (Figure 2). Finally, in the third study, we replicated the effect of 22R‐hydroxycholesterol on *AKR1D1* expression in that our highest two doses induced expression compared to the control (*F*_3, 36_ = 9.56, *p* = 0.009) (Figure 3).

## 4 Discussion

Results from these studies demonstrate that ligands of LXRα can induce expression of *AKR1D1* in the extraembryonic membranes of chicken embryos. Since 5β‐reductase is involved in the metabolism of yolk testosterone (Paitz et al. 2011), progesterone (Paitz and Cagney 2019), and corticosterone (Harders, Agustin, et al. 2024), LXRα appears to be a vital regulator of embryonic exposure to yolk steroids. Variation in 5β‐reductase levels could alter the amount of yolk steroid available to influence development and subsequently dampen or prevent yolk steroid effects. Variation in 5β‐reductase levels could also alter embryonic exposure to 5β‐reduced metabolites that may influence development differently than the parent steroid (Wang et al. 2024). We have shown that corticosterone induces the expression of acyl‐CoA thioesterase 13 (*Acot13*), but the primary 5β‐reduced metabolite of corticosterone, 5β‐corticosterone (Harders, Agustin, et al. 2024), does not induce *Acot13* expression (Harders, Charboneau, et al. 2024). While identifying LXRα as a regulator of *AKR1D1* expression advances our understanding of how yolk steroid effects are modulated by the embryo, it is clear that more work remains to be done on this topic.

The concept of yolk steroids having pleiotropic effects with other egg components is not novel (Groothuis and Schwabl 2008). Our results add ligands of LXRα to the list of egg components that could influence the effects of yolk steroids. For example, yolk testosterone may be more likely to exert an effect in eggs that do not have high levels of LXRα ligands. Similarly, competitive inhibition, where one steroid slows the metabolism of another steroid, could alter embryonic steroid exposure. For example, elevated progesterone levels may slow the metabolism of testosterone or corticosterone as 5β‐reductase is occupied converting progesterone to pregnenedione at the expense of testosterone and corticosterone metabolism. It is becoming clearer how yolk steroids might affect other yolk steroids to alter their respective effects. From an experimental standpoint, studies that manipulate multiple steroids simultaneously are necessary to understand their potential pleiotropic interactions. Some studies have done this within a class of steroids (i.e., multiple androgens) (Saino et al. 2006), but most have manipulated one steroid at a time (Mentesana et al. 2025).

When considering the endogenous ligands for LXRα, such as 22R‐hydroxycholesterol, these oxysterols are not typically considered steroids (Javitt 2008). However, the primary enzyme responsible for producing 22R‐hydroxycholesterol from cholesterol is Cytochrome P450 11A1 (also known as cholesterol side chain cleavage/P450scc) (Tuckey 1992). This is the same enzyme responsible for converting cholesterol to pregnenolone in the initial step of steroid biosynthesis (Schiffer et al. 2019; Dickson et al. 2022). This creates a situation where cholesterol is potentially serving as a substrate for bile acid production (via *AKR1D1)*, oxysterol production, or steroid production during the early stages of embryonic development, and variation in cholesterol dynamics is likely to have implications for the developing embryo. To our knowledge, levels of oxysterols in bird eggs have never been quantified, but levels of 22R‐hydroxycholesterol are elevated during fetal development in humans (Dickson et al. 2022). Our findings that ligands of LXRα induce *AKR1D1* expression early in development advance our understanding of how cholesterol and steroid metabolism are regulated and can be used to help decipher how maternally derived steroids in bird eggs may (or may not) affect offspring development.

## Author Contributions

Conceptualization: Ryan T. Paitz and Emily P. Harders. Data curation: Ryan T. Paitz. Formal analysis: Ryan T. Paitz. Funding acquisition: Ryan T. Paitz and Delaney K. Reynolds. Investigation: Sara E. Waters, Delaney K. Reynolds, Emily M. Drew, and Emily P. Harders. Methodology: Sara E. Waters, Delaney K. Reynolds, Emily M. Drew, and Emily P. Harders. Supervision: Ryan T. Paitz and Emily P. Harders. Writing the original draft: Ryan T. Paitz. Writing, review, and editing: Ryan T. Paitz and Emily P. Harders.

## Conflicts of Interest

The authors declare no conflicts of interest.

## Supporting information

LXR paper datafile.xlsx.

## Data Availability

All relevant data and resources can be found within the article and its supplementary information.

## References

[jez70078-bib-0001] Baggott, G. K. 2001. “Development of Extra‐Embryonic Membranes and Fluid Compartments.” In Perspectives in Fertilisation and Embryonic Development in Poultry, edited by D. C. Deeming , 23–29. Ratite Conference Books.

[jez70078-bib-0002] Balthazart, J. , and D. Hirschberg . 1979. “Testosterone Metabolism and Sexual Behavior in the Chick.” Hormones and Behavior 12, no. 3: 253–263. 546710 10.1016/0018-506x(79)90008-4

[jez70078-bib-0003] Barker, D. J. P. 1995. “Fetal Origins of Coronary Heart Disease.” BMJ 311, no. 6998: 171–174. 7613432 10.1136/bmj.311.6998.171 PMC2550226

[jez70078-bib-0004] Barnett, C. A. , S. G. Clairardin , C. F. Thompson , and S. K. Sakaluk . 2011. “Turning a Deaf Ear: A Test of the Manipulating Androgens Hypothesis in House Wrens.” Animal Behaviour 81, no. 1: 113–120.

[jez70078-bib-0005] Campbell, N. A. , R. Angles , R. M. Bowden , J. M. Casto , and R. T. Paitz . 2020. “Characterizing the Timing of Yolk Testosterone Metabolism and the Effects of Etiocholanolone on Development in Avian Eggs.” Journal of Experimental Biology 223, no. 4: jeb210427. 32001543 10.1242/jeb.210427

[jez70078-bib-0006] Chen, M. , and T. M. Penning . 2014. “5β‐Reduced Steroids and Human Δ4‐3‐Ketosteroid 5β‐Reductase (AKR1D1).” Steroids 83: 17–26. 24513054 10.1016/j.steroids.2014.01.013 PMC3971473

[jez70078-bib-0007] Clairardin, S. G. , C. A. Barnett , S. K. Sakaluk , and C. F. Thompson . 2011. “Experimentally Increased in Ovo Testosterone Leads to Increased Plasma Bactericidal Activity and Decreased Cutaneous Immune Response in Nestling House Wrens.” Journal of Experimental Biology 214, no. 16: 2778–2782. 21795576 10.1242/jeb.054833

[jez70078-bib-0008] Dickson, A. L. , E. Yutuc , C. A. Thornton , Y. Wang , and W. J. Griffiths . 2022. “Identification of Unusual Oxysterols Biosynthesised in Human Pregnancy by Charge‐Tagging and Liquid Chromatography‐Mass Spectrometry.” Frontiers in Endocrinology 13: 1031013. 36440193 10.3389/fendo.2022.1031013 PMC9685423

[jez70078-bib-0009] Eising, C. M. , W. Müller , C. Dijkstra , and T. G. G. Groothuis . 2003. “Maternal Androgens in Egg Yolks: Relation With Sex, Incubation Time and Embryonic Growth.” General and Comparative Endocrinology 132, no. 2: 241–247. 12812771 10.1016/s0016-6480(03)00090-x

[jez70078-bib-0010] Elf, P. K. , and A. J. Fivizzani . 2002. “Changes in Sex Steroid Levels in Yolks of the Leghorn Chicken, *Gallus domesticus*, During Embryonic Development.” Journal of Experimental Zoology 293, no. 6: 594–600. 12410608 10.1002/jez.10169

[jez70078-bib-0011] Enos, J. K. , R. Ducay , R. T. Paitz , M. P. Ward , and M. E. Hauber . 2023. “Female Red‐Winged Blackbirds (*Agelaius phoeniceus*) Do Not Alter Nest Site Selection, Maternal Programming, or Hormone‐Mediated Maternal Effects in Response to Perceived Nest Predation or Brood Parasitism Risk.” General and Comparative Endocrinology 341: 114322. 37247827 10.1016/j.ygcen.2023.114322

[jez70078-bib-0012] Groothuis, T. G. G. , W. Müller , N. von Engelhardt , C. Carere , and C. Eising . 2005. “Maternal Hormones as a Tool to Adjust Offspring Phenotype in Avian Species.” Neuroscience and Biobehavioral Reviews 29, no. 2: 329–352. 15811503 10.1016/j.neubiorev.2004.12.002

[jez70078-bib-0013] Groothuis, T. G. G. , B. Y. Hsu , N. Kumar , and B. Tschirren . 2019. “Revisiting Mechanisms and Functions of Prenatal Hormone‐Mediated Maternal Effects Using Avian Species as a Model.” Philosophical Transactions of the Royal Society, B: Biological Sciences 374, no. 1770: 20180115. 10.1098/rstb.2018.0115 PMC6460091 30966885

[jez70078-bib-0014] Groothuis, T. G. G. , and H. Schwabl . 2008. “Hormone‐Mediated Maternal Effects in Birds: Mechanisms Matter but What Do We Know of Them?” Philosophical Transactions of the Royal Society, B: Biological Sciences 363, no. 1497: 1647–1661. 10.1098/rstb.2007.0007 PMC2606725 18048291

[jez70078-bib-0015] Hamburger, V. , and H. L. Hamilton . 1951. “A Series of Normal Stages in the Development of the Chick Embryo.” Journal of Morphology 88, no. 1: 49–92. 24539719

[jez70078-bib-0016] Harders, E. P. , M. Agustin , and R. T. Paitz . 2024. “Avian Extraembryonic Membranes Respond to Yolk Corticosterone Early in Development.” Biology Open 13, no. 1: bio060131. 38156650 10.1242/bio.060131 PMC10836647

[jez70078-bib-0017] Harders, E. P. , C. Charboneau , and R. T. Paitz . 2024. “Extraembryonic Metabolism of Corticosterone Protects Against Effects of Exposure.” General and Comparative Endocrinology 347: 114439. 38158163 10.1016/j.ygcen.2023.114439

[jez70078-bib-0018] Hauber, M. E. , M. Abolins‐Abols , C. R. Kim , and R. T. Paitz . 2020. “Inter‐Individual Variation in Anti‐Parasitic Egg Rejection Behavior: A Test of the Maternal Investment Hypothesis.” Integrative Organismal Biology (Oxford, England) 2, no. 1: 014. 10.1093/iob/obaa014 PMC7671127 33791557

[jez70078-bib-0019] Henriksen, R. , S. Rettenbacher , and T. G. Groothuis . 2011. “Prenatal Stress in Birds: Pathways, Effects, Function and Perspectives.” Neuroscience and Biobehavioral Reviews 35, no. 7: 1484–1501. 21536067 10.1016/j.neubiorev.2011.04.010

[jez70078-bib-0020] Herbst, A. L. , H. Ulfelder , and D. C. Poskanzer . 1971. “Adenocarcinoma of the Vagina: Association of Maternal Stilbestrol Therapy With Tumor Appearance in Young Women.” New England Journal of Medicine 284, no. 16: 878–881. 5549830 10.1056/NEJM197104222841604

[jez70078-bib-0021] Janowski, B. A. , M. J. Grogan , S. A. Jones , et al. 1999. “Structural Requirements of Ligands for the Oxysterol Liver X Receptors LXRα and LXRβ.” Proceedings of the National Academy of Sciences of the United States of America 96, no. 1: 266–271. 9874807 10.1073/pnas.96.1.266 PMC15128

[jez70078-bib-0052] Javitt, N. B. 2008. “Oxysterols: Novel Biologic Roles for the 21st Century.” Steroids 73, no. 2: 149–157. 18068744 10.1016/j.steroids.2007.10.004

[jez70078-bib-0022] Kumar, N. , A. van Dam , H. Permentier , et al. 2019. “Avian Yolk Androgens Are Metabolized Rather Than Taken Up by the Embryo During the First Days of Incubation.” Journal of Experimental Biology 222, no. 7. 10.1242/jeb.193961 30862703

[jez70078-bib-0023] Kumar, N. , M. van Faassen , I. Kema , M. Gahr , and T. G. G. Groothuis . 2018. “Early Embryonic Modification of Maternal Hormones Differs Systematically Among Embryos of Different Laying Order: A Study in Birds.” General and Comparative Endocrinology 269: 53–59. 30110617 10.1016/j.ygcen.2018.08.014

[jez70078-bib-0024] Kumar, N. , A. Lohrentz , M. Gahr , and T. G. Groothuis . 2019. “Steroid Receptors and Their Regulation in Avian Extraembryonic Membranes Provide a Novel Substrate for Hormone Mediated Maternal Effects.” Scientific Reports 9, no. 1: 11501. 31395925 10.1038/s41598-019-48001-x PMC6687743

[jez70078-bib-0025] Lee, H. C. , Y. Fadaili , and C. D. Stern . 2025. “Development and Functions of the Area Opaca of the Chick Embryo.” Developmental Biology 519: 13–20. 39662721 10.1016/j.ydbio.2024.12.002 PMC11785533

[jez70078-bib-0026] Livak, K. J. , and T. D. Schmittgen . 2001. “Analysis of Relative Gene Expression Data Using Real‐Time Quantitative PCR and the 2− ΔΔCT Method.” Methods 25, no. 4: 402–408. 11846609 10.1006/meth.2001.1262

[jez70078-bib-0027] Massa, R. , L. Cresti , and L. Martini . 1977. “Metabolism of Testosterone in the Anterior Pituitary Gland and the Central Nervous System of the European Starling (*Sturnus vulgaris*).” Journal of Endocrinology 75, no. 3: 347–354. 591837 10.1677/joe.0.0750347

[jez70078-bib-0028] Mentesana, L. , M. Hau , P. B. D'Amelio , N. M. Adreani , and A. Sánchez‐Tójar . 2025. “Do Egg Hormones Have Fitness Consequences in Wild Birds? A Systematic Review and Meta‐Analysis.” Ecology Letters 28, no. 3: e70100. 40102945 10.1111/ele.70100 PMC11920385

[jez70078-bib-0029] Merrill, L. , S. J. Chiavacci , R. T. Paitz , and T. J. Benson . 2017. “Rates of Parasitism, but Not Allocation of Egg Resources, Vary Among and Within Hosts of a Generalist Avian Brood Parasite.” Oecologia 184: 399–410. 28429139 10.1007/s00442-017-3870-z

[jez70078-bib-0030] Merrill, L. , S. J. Chiavacci , R. T. Paitz , and T. J. Benson . 2019. “Quantification of 27 Yolk Steroid Hormones in Seven Shrubland Bird Species: Interspecific Patterns of Hormone Deposition and Links to Life History, Development, and Predation Risk.” Canadian Journal of Zoology 97, no. 1: 1–12.

[jez70078-bib-0031] Muriel, J. , L. Perez‐Rodriguez , M. Puerta , and D. Gil . 2015. “Diverse Dose–Response Effects of Yolk Androgens on Embryo Development and Nestling Growth in a Wild Passerine.” Journal of Experimental Biology 218, no. 14: 2241–2249. 25987739 10.1242/jeb.118257

[jez70078-bib-0032] Navara, K. J. , G. E. Hill , and M. T. Mendonça . 2005. “Variable Effects of Yolk Androgens on Growth, Survival, and Immunity in Eastern Bluebird Nestlings.” Physiological and Biochemical Zoology 78, no. 4: 570–578. 15957111 10.1086/430689

[jez70078-bib-0033] Paitz, R. T. , R. Angles , and E. Cagney . 2020. “In Ovo Metabolism of Estradiol to Estrone Sulfate in Chicken Eggs: Implications for How Yolk Estradiol Influences Embryonic Development.” General and Comparative Endocrinology 287: 113320. 31715137 10.1016/j.ygcen.2019.113320

[jez70078-bib-0034] Paitz, R. T. , R. M. Bowden , and J. M. Casto . 2011. “Embryonic Modulation of Maternal Steroids in European Starlings (*Sturnus vulgaris*).” Proceedings of the Royal Society B: Biological Sciences 278, no. 1702: 99–106. 10.1098/rspb.2010.0813 PMC2992716 20667883

[jez70078-bib-0035] Paitz, R. T. , and E. Cagney . 2019. “In Ovo Metabolism of Progesterone to 5β‐Pregnanedione in Chicken Eggs: Implications for How Yolk Progesterone Influences Embryonic Development.” General and Comparative Endocrinology 282: 113221. 31301283 10.1016/j.ygcen.2019.113221

[jez70078-bib-0036] Paitz, R. T. , and J. M. Casto . 2012. “The Decline in Yolk Progesterone Concentrations During Incubation Is Dependent on Embryonic Development in the European Starling.” General and Comparative Endocrinology 176, no. 3: 415–419. 22210246 10.1016/j.ygcen.2011.12.014

[jez70078-bib-0037] Phoenix, C. H. , R. W. Goy , A. A. Gerall , and W. C. Young . 1959. “Organizing Action of Prenatally Administered Testosterone Propionate on the Tissues Mediating Mating Behavior in the Female Guinea Pig.” Endocrinology 65, no. 3: 369–382. 14432658 10.1210/endo-65-3-369

[jez70078-bib-0038] Podmokła, E. , S. M. Drobniak , and J. Rutkowska . 2018. “Chicken or Egg? Outcomes of Experimental Manipulations of Maternally Transmitted Hormones Depend on Administration Method–A Meta‐Analysis.” Biological Reviews 93, no. 3: 1499–1517. 29573376 10.1111/brv.12406

[jez70078-bib-0039] Russell, D. W. 2003. “The Enzymes, Regulation, and Genetics of Bile Acid Synthesis.” Annual Review of Biochemistry 72, no. 1: 137–174. 10.1146/annurev.biochem.72.121801.161712 12543708

[jez70078-bib-0040] Saino, N. , R. P. Ferrari , M. Romano , et al. 2006. “Maternal Allocation of Androgens and Antagonistic Effects of Yolk Androgens on Sons and Daughters.” Behavioral Ecology 17, no. 2: 172–181.

[jez70078-bib-0041] Schiffer, L. , L. Barnard , E. S. Baranowski , et al. 2019. “Human Steroid Biosynthesis, Metabolism and Excretion Are Differentially Reflected by Serum and Urine Steroid Metabolomes: A Comprehensive Review.” Journal of Steroid Biochemistry and Molecular Biology 194: 105439. 31362062 10.1016/j.jsbmb.2019.105439 PMC6857441

[jez70078-bib-0042] Schultz, J. R. , H. Tu , A. Luk , et al. 2000. “Role of LXRs in Control of Lipogenesis.” Genes & Development 14, no. 22: 2831–2838. 11090131 10.1101/gad.850400 PMC317060

[jez70078-bib-0043] Sharp, P. J. , and R. Massa . 1980. “Conversion of Progesterone to 5α‐and 5β‐Reduced Metabolites in the Brain of the Hen and Its Potential Role in the Induction of the Preovulatory Release of Luteinizing Hormone.” Journal of Endocrinology 86, no. 3: 459–464. 7430904 10.1677/joe.0.0860459

[jez70078-bib-0044] Sheriff, M. J. , A. Bell , R. Boonstra , et al. 2017. “Integrating Ecological and Evolutionary Context in the Study of Maternal Stress.” Integrative and Comparative Biology 57, no. 3: 437–449. 28957523 10.1093/icb/icx105 PMC5886325

[jez70078-bib-0045] Soma, K. K. , B. A. Schlinger , J. C. Wingfield , and C. J. Saldanha . 2003. “Brain Aromatase, 5α‐Reductase, and 5β‐Reductase Change Seasonally in Wild Male Song Sparrows: Relationship to Aggressive and Sexual Behavior.” Journal of Neurobiology 56, no. 3: 209–221. 12884261 10.1002/neu.10225

[jez70078-bib-0046] Tuckey, R. C. 1992. “Cholesterol Side‐Chain Cleavage by Mitochondria From the Human Placenta. Studies Using Hydroxycholesterols as Substrates.” Journal of Steroid Biochemistry and Molecular Biology 42, no. 8: 883–890. 1525048 10.1016/0960-0760(92)90097-3

[jez70078-bib-0047] Vassallo, B. G. , H. P. Litwa , M. F. Haussmann , and R. T. Paitz . 2019. “In Ovo Metabolism and Yolk Glucocorticoid Concentration Interact to Influence Embryonic Glucocorticoid Exposure Patterns.” General and Comparative Endocrinology 272: 57–62. 30500372 10.1016/j.ygcen.2018.11.013 PMC7480747

[jez70078-bib-0048] Vassallo, B. G. , R. T. Paitz , V. J. Fasanello , and M. F. Haussmann . 2014. “Glucocorticoid Metabolism in the in Ovo Environment Modulates Exposure to Maternal Corticosterone in Japanese Quail Embryos (*Coturnix japonica*).” Biology Letters 10, no. 11: 20140502. 25392311 10.1098/rsbl.2014.0502 PMC4261852

[jez70078-bib-0049] von Engelhardt, N. , R. Henriksen , and T. G. G. Groothuis . 2009. “Steroids in Chicken Egg Yolk: Metabolism and Uptake During Early Embryonic Development.” General and Comparative Endocrinology 163, no. 1–2: 175–183. 19362557 10.1016/j.ygcen.2009.04.004

[jez70078-bib-0050] Wang, Y. , B. Riedstra , M. van Faassen , A. Pranger , I. Kema , and T. G. G. Groothuis . 2023. “Dynamics of Maternal Androgens and Its Metabolites During Early Embryonic Development: Embryonic Modification of a Maternal Effect.” Journal of Endocrinology 258, no. 2. 10.1530/JOE-22-0299 37161994

[jez70078-bib-0051] Wang, Y. , B. Riedstra , and T. Groothuis . 2024. “Effects of Maternal Androgens and Their Metabolite Etiocholanolone on Prenatal Development in Birds.” Journal of Experimental Biology 227, no. 15: jeb247205. 39037123 10.1242/jeb.247205 PMC11418167

